# Hospitalizations and surgical management of lumbar disc degeneration in Italy: a 22-Year nationwide retrospective study

**DOI:** 10.1186/s12891-025-09317-0

**Published:** 2025-11-19

**Authors:** Fabrizio Russo, Bruna Maria Rondinone, Giuseppe Francesco Papalia, Gianluca Vadalà, Rocco Papalia, Vincenzo Denaro, Sergio Iavicoli

**Affiliations:** 1 Research Unit of Orthopaedic and Trauma Surgery, Department of Medicine and Surgery, Università Campus Bio-Medico di Roma, Via Alvaro del Portillo 21, Rome, 00128 Italy; 2 Operative Research Unit of Orthopaedic and Trauma Surgery, Fondazione Policlinico Universitario Campus Bio-Medico, Via Alvaro del Portillo 200, Rome, 00128 Italy; 3 Department of Medicine, Epidemiology, Workplace and Environmental Hygiene, Italian Workers’ Compensation Authority (INAIL), Monteporzio Catone (RM), Via Fontana Candida, 1, Monteporzio Catone (RM), 1 - 00078 Italy; 4 Directorate-General for Communication, Ministry of Health, Lungotevere Ripa, 1, Rome, 00153 Italy

**Keywords:** Lumbar disc degeneration, Lumbar spine surgery, Lumbar discectomy, Lumbar spinal fusion, Nationwide analysis, Spinal surgery trends, Standardized hospitalization rate, Hospital discharge data

## Abstract

**Background:**

Lumbar spine disorders are a major contributor to disability and healthcare utilization worldwide, particularly in aging populations. Over the past two decades, significant changes have occurred in surgical techniques for managing lumbar spine disorders. This study aims to provide a comprehensive analysis of hospitalization trends for lumbar disc herniation and intervertebral disc degeneration in the Italian population from 2001 to 2022.

**Methods:**

This nationwide retrospective study used data from the national hospital discharge (NHD) database managed by the Italian Ministry of Health. All patients hospitalized for surgical treatment of lumbar disc degeneration during the 22-year period were included. Age- and sex- standardized hospitalization rates (SHRs) were calculated using direct standardization. Poisson regression identified factors associated with length of hospital stay. Interregional mobility was analyzed as the percentage of hospitalizations in each region relative to its resident population.

**Results:**

Between 2001 and 2022, 621,948 patients (57.4% male, 42.9% aged 45–64 years) underwent surgical treatment for lumbar disc degeneration. Age-sex SHRs declined from 57.41 to 49.43 per 100,000, peaking at 66.38 in 2004. Average hospital stay decreased from 6.4 in 2001 to 3.0 days in 2022. Longer stays were observed in patients ≥ 75 years (IRR = 1.070; 95% CI: 1.011–1.132), in Southern Italy (IRR = 1.199; 95% CI: 1.174–1.225) or the Islands (IRR = 1.165; 95% CI: 1.136–1.195), and in patients undergoing fusion surgery (IRR = 1.905; 95% CI: 1.873–1.938). Admission to private institutions was associated with 25.7% shorter stays (IRR = 0.743, 95% CI: 0.726–0.755). Decompression procedures peaked at > 32,000 cases in 2004 and declined to around 20,000 by 2022, fusion surgeries steadily increased to > 5,700 cases, and arthroplasty, introduced in 2009, remained < 60 cases annually. Several regions reported > 80% of residents hospitalized locally, along with substantial interregional patient flows.

**Conclusions:**

This study provides a comprehensive overview of lumbar spine management trends in Italy. Over 22 years, surgeries shifted from decompression to fusion, hospital stays decreased, and length of stay was influenced by age, region, surgical type, and institution. Regional disparities and patient mobility reflect differences in healthcare access. These findings highlight evolving surgical practices and can inform future planning in spinal care management.

**Supplementary Information:**

The online version contains supplementary material available at 10.1186/s12891-025-09317-0.

## Background

Lumbar spine disorders, including lumbar disc herniation (LDH), lumbar discopathy, and intervertebral disc degeneration (IVDD) contribute significantly to disability and healthcare utilization worldwide [1], particularly within aging populations such as that of Italy. Lumbar IVDD is a multifactorial process caused by genetic, environmental, and biomechanical factors, with presentations ranging from asymptomatic to severe morbidity as the disease progresses. Lumbar spine disorders not only cause considerable discomfort and functional limitations, but also result in significant economic burdens due to frequent hospital admissions, surgical treatments, and long-term rehabilitation [2]. The prevalence and impact of these disorders are likely to increase as the average age of the population rises, further exacerbating the significant public health challenges associated with musculoskeletal disorders [3]. Furthermore, the economic burden associated with these conditions is substantial, including both direct medical costs, and indirect costs related to years lived with disability (YLD), or more commonly increased absenteeism from work due to sick leave and delayed return to work [4, 5]. Over the past two decades, there has been a notable change in the types of surgeries performed and technologies used to treat lumbar spine disorders. Lumbar fusion has increasingly become a prominent surgical approach for degenerative disc disease and has been associated with significant variations in clinical practice [6]. Moreover, the spread of minimally invasive spine surgery (MISS), along with navigated and robotic systems, has significantly transformed the management of these conditions [7]. These innovations have improved surgical precision, reduced blood loss and hospital stays, improved patient clinical outcomes and decreased the complications associated with traditional surgical techniques [8]. Therefore, as these conditions are leading causes of chronic pain and disability, it is crucial to understand the trends of lumbar spine disorders. This study aims to provide a comprehensive analysis of hospitalization trends for lumbar disc herniation and lumbar intervertebral disc degeneration in the Italian population from 2001 to 2022. Leveraging data from the Italian Ministry of Health’s national hospital discharge (NHD) reports, this study examines the demographic characteristics, standardized hospitalization rates, interregional mobility, and changes in treatment modalities associated with these disorders over a 22-year period. Our hypotheses were that the development of new technologies and minimally invasive techniques has contributed to the increase in fusion procedures, and regional differences have influenced both hospitalization rates and interregional mobility. Our results may provide valuable insights into the impact of these spinal disorders on healthcare resources in Italy and contribute to the development of future health strategies for their management and prevention.

## Materials and methods

This retrospective study was based on data collected from NHD database managed by the Italian Ministry of Health. The NHD database is designed to collect information on all hospitalizations in public and private care settings across Italy. It includes details about the patient’s personal characteristics (e.g., age, gender, region of residence, and level of education), the characteristics of hospitalization (e.g., institution, discharge discipline, type of hospitalization, method of discharge, booking date), and clinical information (e.g., main diagnosis, concomitant diagnoses, and diagnostic or therapeutic procedures). Diagnoses and procedures are coded according to the International Classification of Diseases, Ninth Revision, Clinical Modification (ICD-9-CM) [9]. Due to the comprehensive nature of the data collected, the NHD has become an essential tool for various types of analysis, extending beyond the purely administrative purposes for which it was originally designed [10].

This study was conducted using fully anonymized administrative data from the NHD database of the Italian Ministry of Health and did not involve any direct experimentation or interaction with human subjects, nor the use of identifiable personal information. Therefore, according to Italian legislation, ethical review and approval were not required.

Specifically, Decree Law No. 158/2012 (converted into Law No. 189/2012), together with the Ministerial Decree of February 8, 2013, defines the responsibilities of ethics committees in Italy as pertaining to clinical trials of medicinal products, studies involving medical devices, surgical or clinical procedures, or research on food products involving human subjects. As this study is based solely on anonymized administrative data and does not fall into any of these categories, it is exempt from ethical review.

Moreover, the study complies with the General Authorization for the Processing of Personal Data for Scientific Research Purposes, issued by the Italian Data Protection Authority (Authorization No. 9/2014), which permits the use of fully anonymized data without requiring informed consent. Given the nature of the data and the study design, the requirement for informed consent was also waived in accordance with national legislation.

This study was conducted in accordance with the ethical standards of institutional and national research governance and with the principles of the 1964 Declaration of Helsinki and its later amendments.

### Criteria of inclusion and exclusion

The study population included patients aged $\:\ge\:$15 years who were hospitalized between January 1, 2001, and December 31, 2022, and discharged with one of the following primary diagnosis or procedure codes, as classified by the ICD-9-CM. The primary diagnosis codes included were: 722.10 (Displacement of lumbar intervertebral disc without myelopathy), 722.52 (Degeneration of lumbar or lumbosacral intervertebral disc), 722.73 (Intervertebral disc disorder with myelopathy, lumbar region), 722.93 (Other and unspecified disc disorder, lumbar region), 724.2 (Lumbago), 724.3 (Sciatica). The procedure codes considered were: 80.50 (Excision or destruction of intervertebral disc, unspecified), 80.51 (Excision of intervertebral disc), 80.59 (Other destruction of intervertebral disc), 81.06 (Lumbar and lumbosacral fusion, anterior technique), 81.07 (Lumbar and lumbosacral fusion, lateral transverse process technique), 81.08 (Lumbar and lumbosacral fusion, posterior technique), 84.58 (Interspinous process decompression device implantation), 84.64 (Insertion of partial spinal disc prosthesis, lumbosacral), 84.65 (Insertion of total spinal disc prosthesis, lumbosacral). Hospitalizations were classified into three groups based on procedure codes: decompression (codes 80.50, 80.51, 80.59), fusion (codes 81.06, 81.07, 81.08, 84.58), and arthroplasty (84.64, 84.65).

### Interregional mobility

Interregional mobility, specifically the relationship between regions of residence and regions of hospitalization, was analyzed by calculating the percentages of hospitalizations for each region relative to its residents. For this analysis, hospitalizations over the entire 22-year period were aggregated to provide an overall picture of patient flows between regions. For example, for Lombardy, this percentage is obtained by dividing the number of hospitalized individuals in each region by the total number of residents in Lombardy. These values are presented in a heatmap, which can be used to explore how patients move between regions for healthcare. A higher percentage of residents hospitalized outside their region of residence may indicate regional disparities in healthcare availability or the presence of specialized healthcare centers. In the case of strong diagonal dominance (darker cells along the diagonal), regions are largely self-sufficient in terms of hospitalization. In contrast, greater color intensity in off-diagonal cells suggests that certain regions are receiving or sending more patients to other regions for care. This visualization is particularly useful for analyzing regional healthcare utilization patterns and understanding how geographical factors influence hospitalization trends. Although aggregating data over 22 years may obscure temporal trends, this approach provides a comprehensive overview of interregional mobility patterns and can guide the identification of regions with persistent healthcare disparities.

### Statistical analysis

Descriptive statistical analysis was conducted to provide detailed information on the trends in hospitalizations in Italy from 2001 to 2022 for spine-related conditions. Age- and sex-standardized hospitalization rates (SHRs) were calculated using the direct standardization method [11], with the 2001 Italian Census Population serving as the reference population [12]. Age–sex standardization is a statistical technique used to eliminate the effects of age and gender differences when comparing hospitalization rates across different populations or within the same population over time. A multivariable analysis was performed on data of year 2022, using a generalized linear model with a Poisson function to assess the association between the length of hospital stay (in days) and several independent variables, including sex (male, female), age group (15–24, 25–44, 45–64, 65–74, 75 and older), geographic area of hospitalization (North-West, North-East, Centre, South, and Islands), type of intervention (decompression, fusion, arthroplasty) and type of institute (public, private). Poisson regression was chosen because hospital stay represents a count of days, a non-negative integer variable with marked right skewness. This approach allows estimation of Incidence Rate Ratios (IRRs) while adjusting for multiple covariates, and results were therefore expressed as IRRs with corresponding 95% confidence intervals (CIs). All statistical analyses were performed using SPSS version 25 (IBM Corp. Released 2017. IBM SPSS Statistics for Windows, Version 25.0. Armonk, NY: IBM Corp.) and Microsoft Excel (2020). A significance level of 5% was applied for all hypothesis tests.

## Results

### Socio-demographics and clinical characteristics

Between 2001 and 2022, a total of 621,948 patients aged $\:\ge\:$15 years were discharged with one of the selected diagnosis and procedure codes in Italy. Of these, 84.5% was discharged with a diagnosis of displacement of lumbar intervertebral disc without myelopathy (diagnosis code: 722.10) and 90.2% underwent decompression procedures (codes 80.50, 80.51, and 80.59). Regarding socio-demographic variables, 57.4% of patients were males, 42.9% were in the age group 45–64 years, and 30.2% were from the Noth-West of Italy. Additionally, 88.5% of the admissions were planned. More than half of the patients (58.8%) were hospitalized for less than 3 days, and nearly all hospitalizations (94.0%) were fully covered by the national health service (NHS) (Table 1). The age distribution varied significantly by gender (*p* < 0,001): males were more prevalent in the 25–44 age group, while females had higher percentages in the 45–64 and 65–74 age groups.

**Table 1:** Main characteristics of hospitalized cases of spine occurred in Italy between 2001 and 2022

Variable	*N*	%
Diagnosis (ICD-9-CM codes)		
Displacement of lumbar intervertebral disc without myelopathy (722.10)	525,299	84.5
Degeneration of lumbar or lumbosacral intervertebral disc (722.52)	50,205	8.1
Intervertebral disc disorder with myelopathy, lumbar region (722.73)	32,736	5.3
Sciatica (724.3)	10,647	1.7
Lumbago (724.2)	2,025	0.3
Other and unspecified disc disorder, lumbar region (722.93)	1,036	0.2
Procedures (ICD-9-CM codes)		
Decompression (80.50, 80.51, and 80.59)	561,130	90.2
Fusion (81.06, 81.07, 81.08, and 84.58)	60,057	9.7
Arthroplasty (84.64, and 84.65)	761	0.1
Sex		
Male	356,869	57.4
Female	265,079	42.6
Age group		
15–24 years	14,935	2.4
25–44 years	233,059	37.5
45–64 years	266,811	42.9
65–74 years	79,581	12.8
75 and more	27,562	4.4
Geographical area of hospitalization		
North-West	187,883	30.2
North-East	137,446	22.1
Centre	125,794	20.2
South	118,891	19.1
Islands	51,934	8.4
Type of institution of hospitalization		
Public institution	340,670	54.8
Private institution	280,829	45.2
Type of admission		
Programmed	505,107	88.5
Urgent	65,645	11.5
Others/unknown	289	0.1
Length of stay		
≤ 3 days	365,905	58.8
> 3 days	256,043	41.2
Burden on the National Health Service (NHS)		
Hospitalization entirely covered by NHS	582,449	94.0
Hospitalization mainly paid by NHS	14,693	2.4
Hospitalization without charges for the NHS	21,060	3.4
Hospitalization paid by the NHS for foreign patients	921	0.1
Other	582	0.1
Total	621,948	100.0

### Age-sex standardized hospitalization rates

**Fig. 1:**
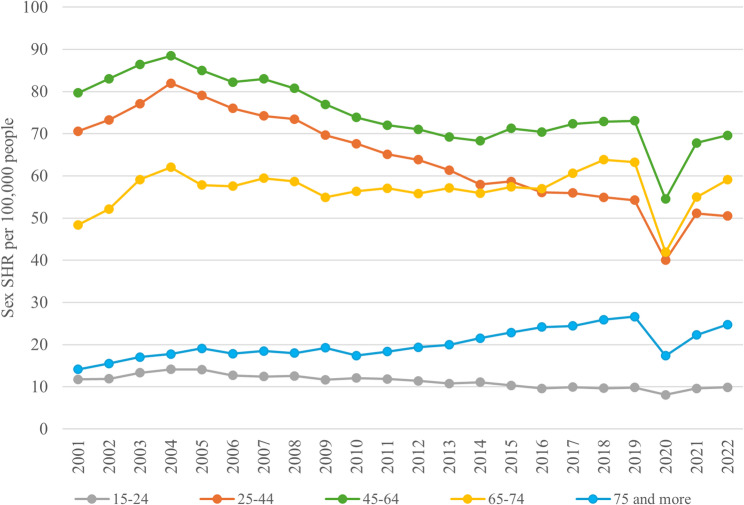
Sex-standardized hospitalization rates (SHRs) per 100,000 people, stratified by age group (Italy, 2001–2022). The 45–64 age group (green) consistently had the highest rates. SHRs in the 25–44 group (orange) declined over time, peaking in 2004. The 65–74 (yellow) group showed an increasing trend throughout the period

During the period from 2001 to 2022, a decreasing trend in age-sex SHRs was observed in the total population, declining from 57.41 per 100,000 people in 2001 to 49.43 per 100,000 people in 2022. A peak was recorded in 2004, with a rate of 66.38 per 100,000 people. This downward trend in SHRs was seen in both males and females, although males consistently showed higher age SHR than females in each year. The male/female ratio also decreased over time but remained greater than 1 throughout the period, ranging from a maximum of 1.56 in 2001 to a minimum of 1.20 in 2022 (Table 2). Higher values of age SHRs were consistently observed in the 45–64 age group over the entire period. The 25–44 age group showed a decreasing trend, with the highest rate recorded in 2004 (81.97 per 100,000 people) and the lowest in 2022 (50.50 per 100,000 people), excluding the anomalous value recorded in 2020 due to the pandemic. In contrast, the 65–74 age group exhibited an increasing trend, starting at 48.40 per 100,000 people in 2001 and rising to 59.09 per 100,000 people in 2022 (Fig. 1).

**Table 2:** Age-sex standardized hospitalization rates per 100,000 people by year

Year	M/F ratio	Age-sex Standardized Hospitalization Rate	Age-Standardized Hospitalization Rates	
			Male	Female
2001	1.56	57.41	73.00	43.07
2002	1.49	59.95	74.79	46.30
2003	1.49	63.48	79.17	49.03
2004	1.46	66.38	82.08	51.90
2005	1.42	63.95	78.25	50.74
2006	1.45	61.71	75.94	48.54
2007	1.40	61.55	74.77	49.31
2008	1.42	60.50	73.93	48.09
2009	1.38	57.56	69.60	46.43
2010	1.36	55.97	67.24	45.58
2011	1.32	54.65	65.05	45.10
2012	1.35	53.79	64.59	43.87
2013	1.28	52.52	61.70	44.05
2014	1.27	51.11	59.61	43.23
2015	1.28	52.46	61.59	43.98
2016	1.25	51.26	59.48	43.62
2017	1.27	52.32	60.90	44.28
2018	1.23	52.61	60.22	45.46
2019	1.23	52.44	60.08	45.25
2020	1.23	38.19	43.68	33.01
2021	1.24	48.34	55.62	41.48
2022	1.20	49.43	55.75	43.42

**Fig. 2:**
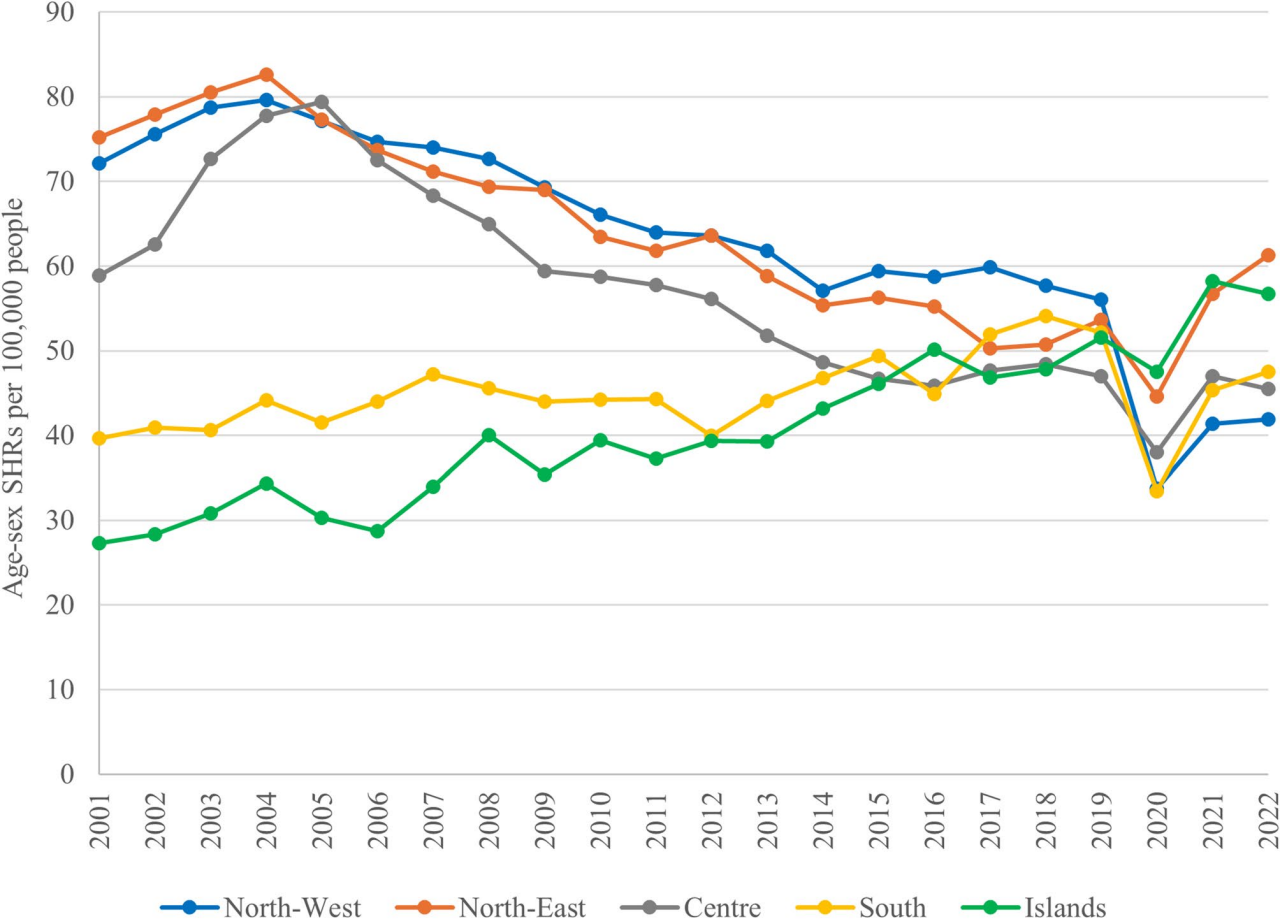
– Age-sex standardized hospitalization rates (SHRs) per 100,000 people, stratified by geographical area of hospitalization (Italy, 2001–2022). A decreasing trend was observed in the North-West (blue), North-East (orange) and Centre (grey). In the South (yellow), SHRs remained consistently lower, while the Islands (green) exhibited an increasing trend throughout the period

Considering the trend of age-sex SHRs by geographical area of hospitalization, a decreasing trend is observed in the North-West, North-East and Centre of Italy. In the South, age-sex SHRs have consistently been lower, ranging between 33.54 per 100,000 people in 2020 and 54.12 per 100,000 people in 2018. In contrast, the Islands exhibited an increasing trend, with rates rising from 27.29 per 100,000 people in 2001 to 56.72 per 100,000 people in 2022 (Fig. 2).

**Fig. 3:**
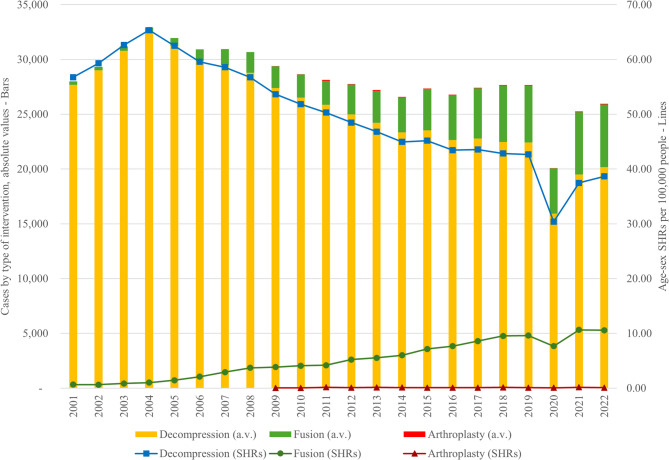
Trends in surgical procedures for spinal conditions, (absolute values - a.v., left axis, bars), and age-sex standardized hospitalization rates (SHRs) per 100,000 people (right axis, lines) in Italy (2001–2022). Decompression (yellow bars) peaked in 2004 before declining, while fusion (green bars) steadily increased. Arthroplasty (red bars), introduced in 2009, remained minimal. SHRs followed a similar pattern, with decompression (blue lines) declining after 2004 and fusion (green lines) gradually increasing

**Fig. 4:**
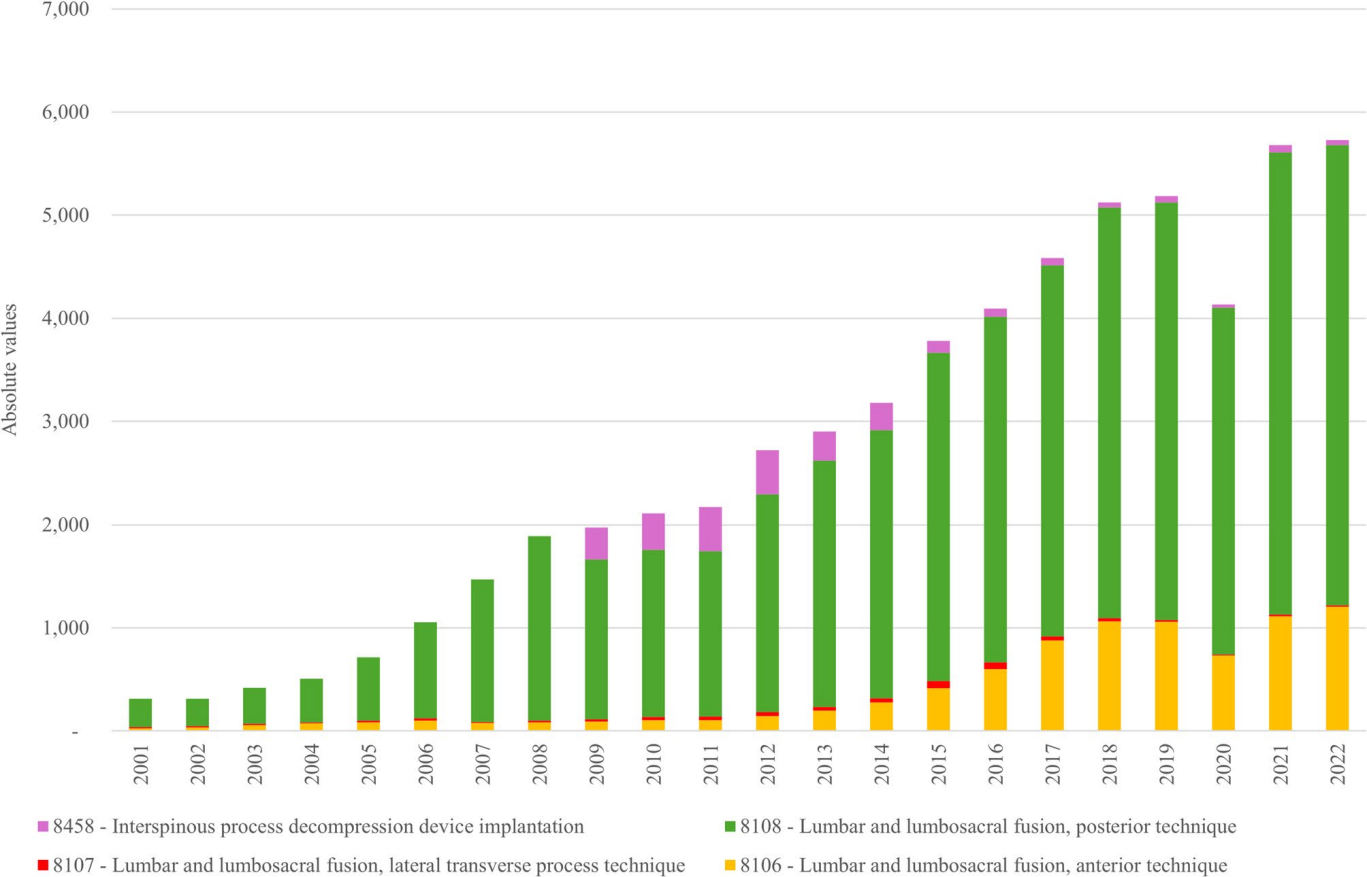
Trends in “fusion” surgical procedures in Italy (2001–2022), absolute values. Posterior fusion (green) remained the most common technique, steadily increasing over time. Anterior fusion (yellow) showed a gradual rise but at lower volumes. The lateral transverse process technique (red) was rare. Interspinous process decompression device implantation (violet) emerged around 2009, peaked in 2011, and then declined sharply

Figure 3, in the left axis (bars), illustrates the trends in surgical procedures for spinal conditions in Italy from 2001 to 2022, categorized into three types: decompression, fusion, and arthroplasty. Decompression procedures, which constitute the largest portion of surgeries, recorded over 27,000 cases in 2002. There was a gradual increase in the following years, reaching a peak of more than 32,000 cases in 2004, followed by a gradual decline to just over 20,000 cases in 2022. Fusion procedures, although consistently fewer than decompression, demonstrated an overall increasing trend throughout the period, reaching more than 5,700 cases by 2022. Arthroplasty, introduced in 2009, represents the smallest proportion of procedures, with fewer than 60 cases annually throughout the period. In the right axis, which depicts the age-sex SHRs per 100,000 people (lines), a similar pattern is observed. The SHR for decompression peaked in 2004 at over 60 per 100,000 people, followed by a steady decline over the years, reaching just above 40 per 100,000 by 2022. Fusion procedures show a slower but consistent upward trend in SHRs, reflecting their increasing frequency, while arthroplasty remains minimal in both cases and hospitalization rates. Overall, the data suggests a general stability in surgical procedures, with some shifts in the types of surgeries performed over the years. Figure 4 illustrates the trend in “fusion” surgery procedures in Italy from 2001 to 2022, categorized into four different types of lumbar and lumbosacral fusion techniques. The posterior fusion technique (code 81.08) dominated the total number of procedures throughout the entire period, showing a steady increase over time, starting with 274 cases in 2001 and reaching over 4,400 cases in 2022. The anterior fusion technique (code 81.06) also showed a gradual rise, starting with 27 cases in 2001 and surpassing 1,200 cases in 2022, though it remained at much lower volumes compared to the posterior technique. The lateral transverse process technique (code 81.07) appeared in very small numbers, with only a few dozen of cases over the period. The interspinous process decompression device implantation (code 84.58) first appeared around 2009 with 308 cases, peaking at 432 cases in 2011, but then declined sharply in subsequent years, reaching only 47 cases by 2022.

### Length of hospital stay and its predictors from Poisson regression analysis

Between 2001 and 2022, the mean hospital length of stay was 4.2 ± 4.1 days (median 3.0), with a clear decreasing trend. In 2001, the average stay was 6.4 ± 6.2 days (median 5.0), while in 2022, it had dropped to 3.0 ± 2.5 days (median 2.0). Although females recorded a slightly higher average length of stay than males throughout the period, both sexes exhibited a similar downward trend. Regarding age groups, a larger difference in mean hospital stay was observed at the start of the period, but this gap diminished over time. In 2001, the mean length of stay was 5.4 ± 4.5 days (median 4.0) for the 15–24 age group and 8.0 ± 8.3 (median 5.0) for those aged 75 and older. By 2022, these values had decreased to 2.7 ± 2.3 (median 2.0) and 3.1 ± 3.2 (median 2.0), respectively. Hospital stays in public institutions ranged from 6.9 ± 6.3 days (median 5.0) in 2001 to 3.1 ± 3.1 days (median 2.0) in 2022. In private institutions, these values 5.4 ± 6.0 (median 4.0) and 2.9 ± 1.9 (median 3.0), respectively. Data on hospital length of stay are described in detail in supplementary material 1.

The Poisson regression model applied to 2022 data revealed that gender was not significantly associated with increased hospital stay (*p* > 0.05). However, length of stay was significantly associated with age group (*p* = 0.001) geographical area of hospitalization (*p* < 0,001), type of intervention (*p* < 0.001) and type of institution (*p* < 0.001). Patients aged 75 years and older were 1.070 times more likely (95% CI: 1.011–1.132) to have a longer hospital stay compared to those aged 15–24 years. Patients from Southern Italy and the Islands had 19.9% (IRR = 1.199; 95% CI: 1.174–1.225) and 16.5% (IRR = 1.165; 95% CI: 1.136–1.195) longer hospital stays, respectively, compared to those from the North-West. Those who underwent fusion surgery were 1.905 times more likely (95% CI: 1.873–1.938) to have a prolonged stay compared to those who underwent decompression surgery. Additionally, being hospitalized in a private institution was associated with a 25.7% shorter length of stay compared to public institutions (IRR = 0.743, 95% CI: 0.726–0.755).

Overall, these findings highlight the significant impact of age, geographical area of hospitalization, type of surgery, and type of institution on length of stay. Older patients, those from Southern Italy and the Islands, those undergoing fusion surgery, and patients in public institutions experienced longer hospital stays (Table 3).

**Table 3:** Poisson regression model results for hospital length of stay

	ß	SE	IRR	CI95%	*p*-value
Gender					
Male	*Ref.*				
Female	0.005	0.0072	1.005	0.991–1.020	0.453
Age group					
15–24	*Ref.*				
25–44	0.018	0.026	1.018	0.966–1.072	0.503
45–64	0.014	0.026	1.014	0.964–1.067	0.595
65–74	0.039	0.027	1.040	0.986–1.096	0.150
75 and more	0.067	0.029	1.070	1.011–1.132	0.019
Geographical area of hospitalization					
North-West	*Ref.*				
North-East	0.047	0.011	1.048	1.026–1.071	< 0.001
Centre	−0.095	0.012	0.910	0.889–0.931	< 0.001
South	0.181	0.011	1.199	1.174–1.225	< 0.001
Islands	0.153	0.013	1.165	1.136–1.195	< 0.001
Type of institution					
Public	*Ref.*				
Private	−0.297	0.008	0.743	0.732–0.755	< 0.001
Type of intervention					
Decompression	*Ref.*				
Fusion	0.644	0.009	1.905	1.873–1.938	< 0.001
Arthroplasty	−0.763	0.113	0.466	0.374–0.581	< 0.001

The model is adjusted for gender, age group, geographical area of hospitalization, type of intervention and type of institutions. The model is based on data collected in 2022.

### Interregional mobility

**Fig. 5:**
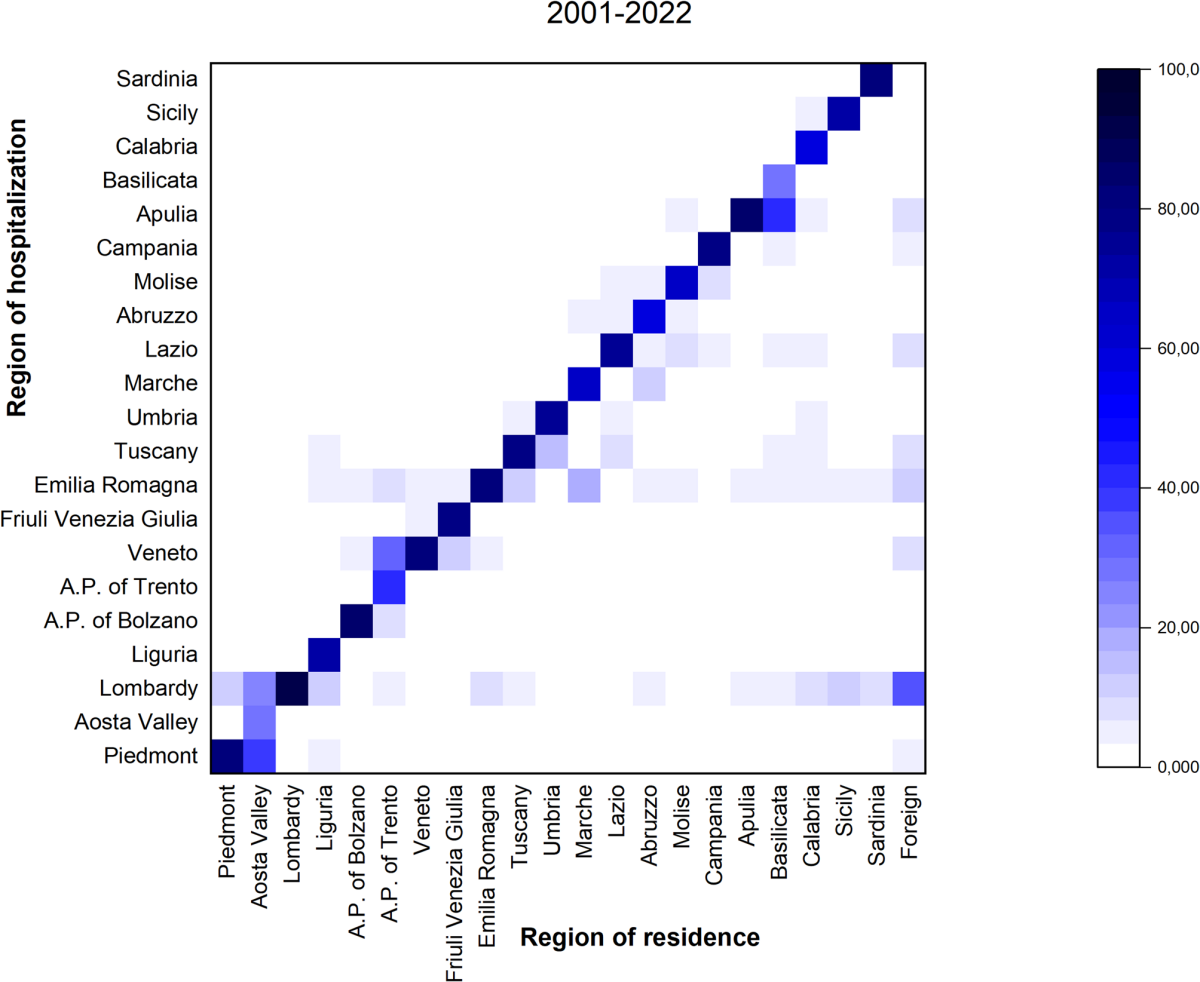
Heatmap of regions of residence and hospitalization in Italy (2001–2022). Percentages of hospitalizations by region (y-axis) relative to residents of each region (x-axis). Darker cells along the diagonal indicate higher percentages of residents hospitalized within their own region, while off-diagonal cells highlight interregional hospitalizations, with darker cells suggesting higher proportions of residents hospitalized in other regions

Figure 5 visualizes interregional mobility, illustrating the relationship between regions of residence (x-axis) and regions of hospitalization (y-axis). The values in each cell represent the percentages of hospitalizations, calculated as the number of hospitalized individuals in the region on the y-axis divided by the number of residents in the region on the x-axis. The darker cells along the diagonal indicate that a large percentage of residents are hospitalized in their own region. Seven regions recorded percentages higher than 80%: Lombardy (91.9%), Autonomous Province of Bolzano (86.5%), Apulia (85.0%), Veneto (82.3%), Sardinia (82.2%), Piedmont (81.4%), and Emilia Romagna (80.8%). In contrast, three regions had percentages at or below 40%: Autonomous Province of Trento (40.7%), Aosta Valley (30.0%), and Basilicata (29.8%). Lighter or darker cells away from the diagonal highlight patterns where residents of one region are hospitalized in a different region. For example, a high percentage of Aosta Valley residents were hospitalized in Piedmont (29.9%) or in Lombardy (24.4%), residents of the Autonomous Province of Trento were hospitalized in Veneto (32.9%), and 42.7% of Basilicata residents in Apulia.

Overall, smaller or less specialized regions showed a marked outflow of patients, primarily directed toward larger neighboring regions with more advanced healthcare facilities. In contrast, northern regions such as Lombardy, Veneto, and Emilia Romagna displayed high levels of self-sufficiency, with the majority of patients treated within their own healthcare systems. These results indicate persistent territorial differences in the distribution of specialized healthcare services.

## Discussion

This study provides a comprehensive analysis of hospitalization trends and surgical treatments for lumbar disc herniation, lumbar discopathy, and lumbar intervertebral disc degeneration in the Italian population over a 22-year period. The primary aim of this study was to assess the evolving patterns of surgical interventions, including decompression, fusion, and arthroplasty, while examining demographic factors, length of hospital stay, and interregional mobility within the healthcare system. To our knowledge, this is one of the most extensive nationwide analyses of lumbar spine disorders conducted in Italy, encompassing over two decades of data and capturing significant trends in both surgical practice and healthcare resource utilization. The data reveal that lumbar spine disorders predominantly affect the middle-aged population, with 42.9% of the hospitalized patients falling within the 45–64 age group. This age distribution is consistent, as degeneration of intervertebral discs tends to increase with age [13]. The study also highlights a notable gender disparity, with males accounting for 57.4% of the total hospitalizations. This higher prevalence among males, particularly in the 25–44 age group, may be associated with greater physical labor demands and occupational exposures contributing to lumbar spine disorders [14, 15]. Conversely, females showed a higher representation in the older age groups. One of the most striking findings of this study is the divergent trends observed in different types of surgical procedures over the 22-year period. Decompression procedures, which have historically been the most common surgical intervention for lumbar spine disorders, showed a marked decrease. From a peak of over 32,000 cases in 2004, decompressions steadily declined to just over 20,000 cases by 2022, with a decrease of 37.5%. Similarly to our findings in Italy, the United States has also seen a significant decline in decompression procedures. In particular, from 2003 to 2013, lumbar discectomies decreased by 19.8%, while lumbar laminectomies declined by 26.1% during the same period [16]. This reduction could be attributed to advancements in non-surgical management and the development of Navigation-Guided/Robot-Assisted MISS [17]. These techniques have been associated with improved accuracy in pedicle screw placement, reduced complications, decreased intraoperative blood loss, and shorter hospital stays, ultimately leading to better long-term outcomes [8, 18]. In a study by Vertuani et al. [19], MISS has been shown to be both a more cost-effective and efficient option compared to open surgery for transforaminal lumbar interbody fusion (TLIF) in Italy and United Kingdom. Moreover, in our analysis lumbar fusions exhibited a significant upward trend, increasing from just over 300 cases in 2001 to more than 5,700 cases by 2022, with an increase of 1,800%. In the United States, a marked increase in lumbar spinal fusion surgeries has been documented, with a trend similar to that observed in our study. A national trend analysis between 1998 and 2008 [20], showed discharges for spinal fusion increased by 137%, while the average age of patients undergoing fusion procedures increased from 48.8 to 54.2 years. To complete this, lumbar spinal fusions in the United States increased by 56.4% between 2003 and 2013, although the overall rate of elective lumbar spine surgeries per 100,000 people slightly decreased [16]. The increase in spinal fusion surgeries, particularly in cases involving spinal instability or deformity, can be attributed to innovations in surgical techniques, such as MISS and advanced implant development. These advancements have made fusion the preferred option in many complex cases where stabilization is needed to restore spinal integrity and alleviate pain [21]. A deeper analysis of fusion techniques reveals additional indications in surgical trends. Posterior lumbar interbody fusion (PLIF), the most frequently performed fusion technique throughout the study period, showed a steady increase, from 274 cases in 2001 to over 4,400 in 2022 (with an increase of 1,506%). This technique remains widely used: the posterior exposure in PLIF provides excellent visualization of the nerve roots, allowing for effective neural decompression while maintaining posterior support structures, and ensuring adequate restoration of interbody height [22]. However, anterior lumbar interbody fusion (ALIF), despite being performed less frequently than PLIF, demonstrated a sharper rise in utilization, increasing from 27 cases in 2001 to over 1,200 in 2022 (with an increase of 4,344%). ALIF is considered the most advantageous techniques for achieving wide fusion, indirect decompression, and restoring physiological lumbosacral height and segmental lordosis; it also allows to preserve the posterior elements and reduce the risk of nerve damage [23, 24]. In contrast, interspinous device implantation reported initial growth after its introduction in 2009 but has since declined dramatically: after peaking at 432 cases in 2011, the number of interspinous device implantations has dropped to just 47 cases in 2022, with a decrease of 89%. This decline was due to a combination of clinical and mechanical concerns, resulting in a high rate of device-related complications, such as dislocation, spinous process fractures, and lack of long-term efficacy, which led to increased rates of revision surgery [25]. Hospitalization length for lumbar spine procedures also displayed significant variation over time. Between 2001 and 2022, the mean hospital stay decreased from 6.4 days to 3.0 days. This improvement can be related to several factors, including advancements in MISS techniques, improved postoperative protocols, and a general trend of shorter hospital stays across all surgical disciplines [26]. The study showed that patients undergoing fusion surgeries had a significantly longer length of stay compared to those undergoing decompression procedures, reflecting the greater complexity and postoperative care associated with fusion [27]. The geographical area of hospitalization also played a role in determining the length of stay, with patients from Southern Italy and the Islands experiencing longer hospitalizations compared to their counterparts in the North. Moreover, the heatmap analysis of interregional mobility provides valuable insights into the healthcare dynamics within Italy. Several regions, such as Lombardy, Emilia Romagna, Apulia and Veneto, demonstrated high self-sufficiency, with over 80% of residents hospitalized within their own region, as well as presenting a significant percentage of hospitalizations of patients residing in other regions. This reflects the presence of well-developed healthcare systems in these areas, capable of providing comprehensive care for lumbar spine disorders. In contrast, regions such as the Autonomous Province of Trento, Aosta Valley, and Basilicata showed much lower self-sufficiency, with a significant proportion of patients seeking care in other regions. The high rates of cross-regional hospitalizations may indicate either a lack of specialized services or regional disparities in healthcare quality and accessibility.

Taken together, these findings may have several important implications. Clinically, they emphasize the growing role of fusion procedures and minimally invasive techniques, underlining the need for continuous surgical training and careful patient selection to ensure appropriate use of advanced interventions. From a research perspective, our results highlight the importance of evaluating long-term outcomes of these evolving surgical practices and assessing their cost-effectiveness in routine care. At the policy level, the observed interregional disparities in patient mobility highlight areas where specialized surgical capacity could be further developed to enhance equity of access and potentially reduce the need for patient migration. In this regard, the study offers insights that may support healthcare planning and guide the allocation of resources within the Italian healthcare system.

This study, while comprehensive and based on a large national dataset, presents some important limitations. As with all analyses based on administrative data, certain inherent limitations should be acknowledged, such as potential coding errors and misclassifications inherent in the NHD database, which could affect the accuracy and reliability of the findings. Although the use of standardized ICD-9-CM codes helps ensure consistency, discrepancies in diagnosis and procedure coding cannot be completely ruled out. Second, the dataset lacks detailed clinical information, such as disease severity, patient comorbidities, or specific indications for surgery, which are critical factors that can influence treatment decisions and outcomes. Third, the retrospective nature of the study, based on pre-existing administrative data, means that important variables, such as postoperative complications and clinical outcomes were unavailable. This limitation restricts the ability to fully assess the clinical effectiveness and safety of the surgical interventions analyzed. Moreover, variations in regional healthcare practices and hospital reporting may also influence the observed trends. Nonetheless, despite these constraints, the study provides valuable epidemiological insights into hospitalization and surgical management trends for lumbar disc degeneration across Italy over an extended period, contributing important information for healthcare planning and policy development.

## Conclusion

The management of lumbar disc degeneration continues to represent a relevant healthcare issue in Italy, often leading to spinal surgery. This national retrospective analysis describes the evolution of epidemiological trends and surgical management of lumbar spine disorders over a 22-year period. The study highlights variations in surgical techniques, regional differences in healthcare access, socio-demographic characteristics of patients, and length of hospital stay. These findings may help inform future evaluations of resource allocation and equity in spine across the country.

## Supplementary Information

Supplementary Material 1.

## Data Availability

The data that support the findings of this study are available from the Italian Ministry of Health, but restrictions apply to the availability of these data, which were used under license for the current study and are therefore not publicly available. Data are, however, available from the authors upon reasonable request and with permission of the Italian Ministry of Health.

## Abbreviations

ALIF: Anterior lumbar interbody fusion
CI: Confidence interval
ICD-9-CM: International Classification of Diseases, Ninth Revision, Clinical Modification
IRR: Incidence rate ratio
IVDD: Intervertebral disc degeneration
LDH: Lumbar disc herniation
MISS: Minimally invasive spine surgery
NHD: National hospital discharge
NHS: National health service
PLIF: Posterior lumbar interbody fusion
SHR: Standardized hospitalization rate
TLIF: Transforaminal lumbar interbody fusion
YLD: Years lived with disability
