# Supplementary material for: Hospitalizations and surgical management of lumbar disc degeneration in Italy: a 22-Year nationwide retrospective study
Source: BMC Musculoskelet Disord. 2025 Nov 19;26:1058. doi: 10.1186/s12891-025-09317-0 (PMC12628820; doi:10.1186/s12891-025-09317-0)
Supplement: Supplementary file 1 — Supplementary Material 1. [file 12891_2025_9317_MOESM1_ESM.docx]

Mean length of stay (M), median (Me), and Standard Deviation (SD) in days by gender, age group and type of institute, Italy, 2001-2022

|  | **Total** | | **Male** | |  | **Female** | | **15-24** | |  | **25-44** | |  | **45-64** | |  | **65-74** | |  | **75 and older** | | **Public institutions** | |  | **Private institutions** | |
| --- | --- | --- | --- | --- | --- | --- | --- | --- | --- | --- | --- | --- | --- | --- | --- | --- | --- | --- | --- | --- | --- | --- | --- | --- | --- | --- |
|  | **M (Me)** | **DS** | **M (Me)** | **DS** |  | **M (Me)** | **DS** | **M (Me)** | **DS** |  | **M (Me)** | **DS** |  | **M (Me)** | **DS** |  | **M (Me)** | **DS** |  | **M (Me)** | **DS** | **M (Me)** | **DS** |  | **M (Me)** | **DS** |
| 2001 | 6.4 (5.0) | 6.2 | 6.4 (5.0) | 6.4 |  | 6.5 (5.0) | 6.0 | 5.4 (4.0) | 4.5 |  | 5.9 (4.0) | 4.9 |  | 6.6 (5.0) | 6.4 |  | 7.9 (5.0) | 9.2 |  | 8.0 (5.0) | 8.3 | 6.9 (5.0) | 6.3 |  | 5.4 (4.0) | 6.0 |
| 2002 | 5.7 (4.0) | 5.1 | 5.6 (4.0) | 4.9 |  | 5.8 (4.0) | 5.3 | 5.0 (4.0) | 4.2 |  | 5.3 (4.0) | 4.3 |  | 5.9 (4.0) | 5.2 |  | 6.6 (4.0) | 6.8 |  | 7.2 (5.0) | 7.0 | 6.1 (4.0) | 5.4 |  | 4.8 (4.0) | 4.2 |
| 2003 | 5.4 (4.0) | 4.8 | 5.3 (4.0) | 4.7 |  | 5.5 (4.0) | 5.0 | 4.8 (4.0) | 4.4 |  | 4.9 (4.0) | 3.9 |  | 5.5 (4.0) | 5.0 |  | 6.4 (4.0) | 6.2 |  | 6.8 (4.0) | 7.2 | 5.8 (4.0) | 5.3 |  | 4.6 (3.0) | 3.8 |
| 2004 | 5.0 (4.0) | 4.6 | 5.0 (4.0) | 4.5 |  | 5.1 (4.0) | 4.8 | 4.4 (3.0) | 3.3 |  | 4.8 (4.0) | 4.1 |  | 5.2 (4.0) | 4.8 |  | 5.6 (4.0) | 5.4 |  | 5.8 (4.0) | 6.5 | 5.4 (4.0) | 5.0 |  | 4.3 (3.0) | 3.7 |
| 2005 | 4.8 (3.0) | 4.3 | 4.7 (3.0) | 4.3 |  | 4.9 (3.0) | 4.3 | 4.3 (3.0) | 3.7 |  | 4.5 (3.0) | 3.7 |  | 4.9 (3.0) | 4.6 |  | 5.3 (4.0) | 5.2 |  | 5.4 (3.0) | 5.1 | 5.2 (4.0) | 4.7 |  | 4.1 (3.0) | 3.5 |
| 2006 | 4.6 (3.0) | 4.3 | 4.5 (3.0) | 4.3 |  | 4.7 (3.0) | 4.4 | 4.1 (3.0) | 3.2 |  | 4.3 (3.0) | 3.9 |  | 4.6 (3.0) | 4.4 |  | 5.2 (3.0) | 5.4 |  | 5.4 (4.0) | 5.4 | 5.0 (4.0) | 4.8 |  | 3.9 (3.0) | 3.5 |
| 2007 | 4.4 (3.0) | 4.1 | 4.3 (3.0) | 4.1 |  | 4.4 (3.0) | 4.1 | 3.8 (3.0) | 3.3 |  | 4.2 (3.0) | 3.6 |  | 4.4 (3.0) | 4.2 |  | 4.8 (3.0) | 5.1 |  | 4.7 (3.0) | 4.8 | 4.8 (3.0) | 4.7 |  | 3.7 (3.0) | 3.1 |
| 2008 | 4.3 (3.0) | 4.0 | 4.2 (3.0) | 3.8 |  | 4.4 (3.0) | 4.2 | 3.9 (3.0) | 3.9 |  | 4.1 (3.0) | 3.5 |  | 4.3 (3.0) | 3.9 |  | 4.8 (3.0) | 5.0 |  | 5.0 (3.0) | 5.5 | 4.8 (3.0) | 4.5 |  | 3.6 (3.0) | 3.1 |
| 2009 | 4.3 (3.0) | 4.0 | 4.2 (3.0) | 3.9 |  | 4.4 (3.0) | 4.2 | 3.7 (3.0) | 2.6 |  | 4.1 (3.0) | 3.4 |  | 4.3 (3.0) | 4.1 |  | 4.8 (3.0) | 5.0 |  | 5.1 (3.0) | 5.9 | 4.8 (3.0) | 4.6 |  | 3.5 (3.0) | 2.9 |
| 2010 | 4.2 (3.0) | 4.0 | 4.1 (3.0) | 3.8 |  | 4.3 (3.0) | 4.1 | 3.7 (3.0) | 2.9 |  | 4.0 (3.0) | 3.5 |  | 4.3 (3.0) | 4.1 |  | 4.6 (3.0) | 4.5 |  | 4.7 (3.0) | 5.4 | 4.9 (4.0) | 4.6 |  | 3.4 (3.0) | 2.8 |
| 2011 | 4.0 (3.0) | 3.8 | 3.9 (3.0) | 3.8 |  | 4.1 (3.0) | 3.9 | 3.7 (3.0) | 3.4 |  | 3.9 (3.0) | 3.3 |  | 4.1 (3.0) | 4.1 |  | 4.2 (3.0) | 4.1 |  | 4.3 (3.0) | 5.0 | 4.8 (3.0) | 4.6 |  | 3.2 (3.0) | 2.5 |
| 2012 | 3.9 (3.0) | 4.0 | 3.8 (3.0) | 3.9 |  | 4.0 (3.0) | 4.2 | 3.6 (3.0) | 2.6 |  | 3.8 (3.0) | 3.9 |  | 3.9 (3.0) | 4.0 |  | 4.1 (3.0) | 4.2 |  | 4.3 (3.0) | 4.8 | 4.6 (3.0) | 4.9 |  | 3.1 (2.0) | 2.5 |
| 2013 | 3.8 (3.0) | 3.5 | 3.7 (3.0) | 3.5 |  | 3.9 (3.0) | 3.6 | 3.5 (3.0) | 3.2 |  | 3.7 (3.0) | 3.1 |  | 3.8 (3.0) | 3.5 |  | 4.0 (3.0) | 4.2 |  | 3.9 (2.0) | 4.6 | 4.5 (3.0) | 4.2 |  | 3.0 (2.0) | 2.4 |
| 2014 | 3.7 (3.0) | 3.5 | 3.6 (3.0) | 3.6 |  | 3.8 (3.0) | 3.5 | 3.5 (3.0) | 4.6 |  | 3.5 (3.0) | 3.0 |  | 3.7 (3.0) | 3.4 |  | 4.0 (3.0) | 4.5 |  | 3.9 (2.0) | 4.8 | 4.3 (3.0) | 4.3 |  | 3.0 (2.0) | 2.4 |
| 2015 | 3.6 (3.0) | 3.3 | 3.6 (3.0) | 3.3 |  | 3.7 (3.0) | 3.4 | 3.3 (3.0) | 2.8 |  | 3.5 (3.0) | 2.9 |  | 3.6 (3.0) | 3.3 |  | 3.7 (3.0) | 3.7 |  | 4.0 (3.0) | 4.6 | 4.1 (3.0) | 4.0 |  | 3.1 (3.0) | 2.3 |
| 2016 | 3.5 (3.0) | 3.3 | 3.5 (3.0) | 3.4 |  | 3.6 (3.0) | 3.3 | 3.2 (3.0) | 2.7 |  | 3.4 (3.0) | 2.8 |  | 3.5 (3.0) | 3.3 |  | 3.8 (3.0) | 4.0 |  | 3.7 (2.0) | 4.1 | 3.9 (3.0) | 4.0 |  | 3.1 (3.0) | 2.4 |
| 2017 | 3.4 (3.0) | 3.1 | 3.4 (3.0) | 3.1 |  | 3.5 (3.0) | 3.1 | 3.0 (2.0) | 2.0 |  | 3.3 (3.0) | 2.7 |  | 3.5 (3.0) | 3.1 |  | 3.6 (3.0) | 3.5 |  | 3.5 (3.0) | 4.0 | 3.8 (3.0) | 3.7 |  | 3.1 (3.0) | 2.3 |
| 2018 | 3.3 (3.0) | 3.0 | 3.3 (3.0) | 2.9 |  | 3.4 (3.0) | 3.1 | 3.0 (2.0) | 2.2 |  | 3.2 (3.0) | 2.5 |  | 3.3 (3.0) | 3.0 |  | 3.4 (3.0) | 3.3 |  | 3.5 (2.0) | 4.3 | 3.6 (2.0) | 3.7 |  | 3.1 (3.0) | 2.2 |
| 2019 | 3.2 (2.0) | 2.9 | 3.2 (2.0) | 2.8 |  | 3.3 (3.0) | 3.0 | 2.7 (2.0) | 2.1 |  | 3.1 (3.0) | 2.5 |  | 3.2 (2.0) | 2.8 |  | 3.4 (2.0) | 3.4 |  | 3.2 (2.0) | 3.5 | 3.4 (2.0) | 3.5 |  | 3.0 (3.0) | 2.1 |
| 2020 | 3.2 (2.0) | 2.9 | 3.1 (2.0) | 2.9 |  | 3.2 (3.0) | 2.8 | 2.8 (2.0) | 2.4 |  | 3.1 (2.0) | 2.3 |  | 3.2 (3.0) | 2.7 |  | 3.3 (2.0) | 3.4 |  | 3.4 (2.0) | 4.7 | 3.6 (3.0) | 3.6 |  | 2.9 (2.0) | 2.2 |
| 2021 | 3.1 (2.0) | 2.5 | 3.1 (2.0) | 2.6 |  | 3.2 (2.0) | 3.2 | 2.7 (2.0) | 1.7 |  | 3.0 (3.0) | 2.3 |  | 3.1 (2.0) | 3.1 |  | 3.3 (2.0) | 3.1 |  | 3.1 (2.0) | 2.8 | 3.4 (2.0) | 3.8 |  | 2.9 (2.0) | 2.0 |
| 2022 | 3.0 (2.0) | 2.5 | 3.0 (2.0) | 2.5 |  | 3.0 (2.0) | 2.5 | 2.7 (2.0) | 2.3 |  | 2.9 (2.0) | 2.0 |  | 3.0 (2.0) | 2.5 |  | 3.1 (2.0) | 3.0 |  | 3.1 (2.0) | 3.2 | 3.1 (2.0) | 3.1 |  | 2.9 (3.0) | 1.9 |
